# Comparing glucose monitoring methods: efficiency insights in a simulated hospital setting

**DOI:** 10.3389/fcdhc.2025.1517161

**Published:** 2025-06-03

**Authors:** Allan Davasgaium, Timothy Robbins, Bianca Leca, Andreea Epure, Sailesh Sankar, Harpal Randeva

**Affiliations:** ^1^ Warwickshire Institute for the Study of Diabetes, Endocrinology and Metabolism (WISDEM), University Hospitals Coventry and Warwickshire NHS Trust, Coventry, United Kingdom; ^2^ Warwick Medical School, University of Warwick, Coventry, United Kingdom; ^3^ Independent Researcher, Oxford, United Kingdom

**Keywords:** continuous glucose monitoring, flash glucose monitoring-dynamic interstitial glucose monitoring, FreeStyle Libre, diabetes, staff perception, capillary blood glucose monitoring, finger-prick test

## Abstract

While the advantages of flash glucose monitoring, also known as dynamic interstitial glucose monitoring (DIGM), are established in outpatient diabetes care, evidence of its impact within hospital settings remains limited. This study compared the efficiency of use and healthcare staff perception of DIGM monitoring versus traditional finger-prick testing in a simulated hospital environment. Twenty-five healthcare professionals (52% nurses, 48% allied healthcare professionals [AHCPs]) participated in simulated clinical scenarios involving glucose monitoring tasks using a high-fidelity mannequin. Participants performed three tasks: (A) applying a flash sensor, (B) scanning the sensor to obtain a glucose reading, and (C) performing a finger-prick test. Task durations and staff perceptions were assessed, with statistical analyses conducted using Python (version 3). DIGM was significantly faster than finger-prick testing. Sensor application took 75.4 ± 22.4 seconds, flash scanning took 26.4 ± 11.5 seconds, and finger-prick testing required 132.8 ± 37 seconds (p < 0.05 for all comparisons). DIGM saved approximately 106 seconds per glucose check based on these timings. Furthermore, a scenario of 20 readings per hospitalized patient translates to an average of 34.2 minutes saved per patient. While staff with greater experience performed tasks slightly faster, the overall time-saving benefit of DIGM remained substantial across all levels of experience. In addition, survey responses revealed a strong staff preference for DIGM, highlighting perceived benefits in workflow efficiency, patient comfort, and infection control. In conclusion, DIGM was significantly more efficient than finger-prick testing and strongly preferred by clinical staff. These technologies offer time-saving benefits that could improve patient experience, streamline clinical workflows, and potentially enhance diabetes management outcomes.

## 1 Introduction

Diabetes is highly prevalent in hospital settings, and dysglycemia in acutely ill patients is linked to worsened outcomes. Approximately one in four patients admitted to the hospital has diabetes (1–3). Inpatient hyperglycemia, whether from pre-existing diabetes or stress responses, is associated with increased risks of complications and mortality, longer hospital stays, and higher rates of intensive care unit admission (2–4). This data underscores the clinical importance of effective glucose monitoring and tight glycemic management in hospitalized patients with diabetes.

For over four decades, the standard of care for inpatient glucose monitoring has been point-of-care capillary blood glucose testing (finger-prick checks) (1). While widely adopted and generally reliable, this traditional approach has significant limitations. Finger-prick monitoring is invasive, painful for patients, and a time-consuming procedure for nursing staff. Increasing finger-prick measurements can improve glycemic control but intensifies workload, making it often impractical. Thus, there is a clear need for monitoring methods that are less labor-intensive yet more informative, supporting tight glycemic control without overburdening staff or patients.

DIGM has emerged as an innovative solution to these challenges in recent years. DIGM uses a sensor applied to the patient’s body to continuously measure interstitial glucose levels, allowing caregivers or patients to obtain readings on demand via a scanner, thereby reducing the need for frequent finger-pricks. Systems like the FreeStyle Libre are proving more effective than traditional finger-prick methods for managing diabetes in hospital settings. These devices help decrease time spent in hypo- or hyperglycemic states (5–8) and substantially reduce the frequency and severity of hypoglycemia in type 2 diabetes patients, with studies showing a decrease of up to 68% in severe cases (7, 9–11).

Furthermore, DIGM detected more nocturnal hypoglycemia episodes in general medicine wards than traditional finger-prick testing, demonstrating superior glucose surveillance without compromising safety (12). Additionally, DIGM offers better glycemic control for type 1 diabetes patients by maintaining consistent blood sugar levels, enhancing the quality of life, and reducing complications (6, 11, 13).

Furthermore, the non-invasive nature of these monitors significantly improves patient satisfaction and comfort, which is particularly valuable in clinical environments. Comprehensive reviews and meta-analyses (7, 8, 12) confirmed the safety and efficacy of these systems, supporting their use in healthcare settings to improve treatment outcomes and overall health in diabetic patients.

In 2022, Robbins et al. (14) documented the initial application of inpatient digital glucose monitoring equipment in a hospital operated by the National Health Service (NHS). Factors such as the duration of hospital stay, HbA1c levels, average glucose levels, and the time spent in different blood sugar states were correlated. Furthermore, DIGM allows more frequent and real-time monitoring of glucose levels, enabling prompt interventions to sustain glucose targets during hospitalization.

Moreover, DIGM has demonstrated acceptable accuracy in inpatient settings. In 2022, Longo et al. assessed DIGM performance among patients in general medicine and intensive care units, comparing readings with point-of-care and laboratory glucose values. The results showed a mean absolute relative difference ranging from 10.9% to 14%, supporting DIGM as an alternative for inpatient glucose monitoring. However, further research is needed to confirm its safety, guide appropriate use, and evaluate features such as trend arrows, alerts, and alarms (15–17).

The COVID-19 pandemic further accelerated this interest in DIGM by highlighting the need to minimize staff exposure. During the pandemic, some hospitals permitted inpatient use of DIGM to reduce healthcare worker contact with patients while maintaining glucose surveillance (15, 18).

Healthcare staff have also reported positive experiences with these systems, noting reduced workload and increased confidence in patient safety (1, 19, 20). Eliminating many routine finger-prick checks saves time, which can be redirected to other patient care tasks, and reduces exposure risk and cross-contamination (21, 22); using a sensor to read glucose noninvasively means fewer open finger-stick wounds and less direct contact with blood, an important safety consideration for both patients and healthcare workers.

Given this growing body of evidence supporting DIGM, further studies are needed to assess its clinical accuracy and impact on patient outcomes and its practical use, operational efficiency, and acceptability among hospital staff, particularly in inpatient settings characterized by high workflow demands. The study aims to address this gap by evaluating the procedural efficiency and staff perceptions of DIGM compared to conventional finger-prick methods within a simulated hospital environment.

## 2 Materials and methods

### 2.1 Study design

A high-fidelity simulation was conducted in a clinical skills laboratory using a mannequin, alongside a mixed-methods survey. Ethical approval was obtained from the Trust’s Ethics Committee (GafREC reference: GF0450). Participants completed simulated hospital scenarios involving three glucose monitoring tasks: (A) applying a flash glucose sensor, (B) scanning with the FreeStyle Libre device, and (C) performing a finger-prick glucose test.

### 2.2 Study population

Participants were recruited from nursing and allied healthcare staff employed within the Diabetes and Endocrinology services at the local Trust. Before participation, written informed consent and approval from line managers were obtained. Eligible participants were familiar with standard ward equipment for finger-prick glucose testing and agreed to receive training in using DIGM.

Participants were informed that the study aimed to evaluate time efficiencies between flash and finger-prick glucose monitoring. They were notified that simulations would be recorded for time analysis and that all video recordings would be anonymized and deleted following data analysis and dissemination.

The study was conducted over four days in the simulation laboratory, with each session lasting approximately one hour per participant.

**Figure 1:**
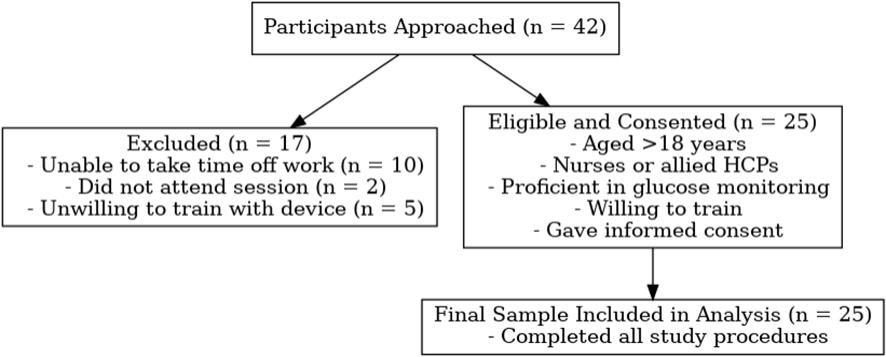
Flow diagram illustrating participant recruitment and inclusion in the simulation-based study.

Of the 42 healthcare staff approached, 25 consented and met the inclusion criteria. Reasons for non-participation included scheduling conflicts (n=10), non-attendance (n=2), and unwillingness to undertake necessary training (n=5). The enrolment process is illustrated in **Figure 1**.

### 2.3 Questionnaires

The survey was administered in paper format immediately after the simulation session, allowing participants to reflect on their experience while it was still recent. The clinical research team developed the instrument internally and reviewed it for clarity and face validity. It consisted of 14 items, comprising 13 Likert-scale questions (a 5-point scale ranging from Strongly Disagree to Strongly Agree) and one open-ended question. The full survey is provided in **Supplementary Data 1**.

All 25 participants completed the Likert-scale items, with a response rate of 100%, while 22 participants (88%) responded to the open-ended question. Two researchers manually entered completed paper surveys into a secure electronic database and cross-verified them to ensure accuracy.

### 2.4 Statistical analysis

Video recordings of the simulation sessions were reviewed, and relevant timings were extracted. These included the time required to set up each device, obtain consent for glucose monitoring, perform the glucose measurement, and complete the procedure, including documentation and appropriate disposal of clinical waste.

Microsoft Excel was utilized for initial data collection and management. All statistical analyses were performed using Python (version 3), employing key libraries such as Pandas (data manipulation), SciPy (statistical testing), Matplotlib and Seaborn (data visualization), and Statsmodels (power analysis).

Depending on the data type and distribution, appropriate statistical tests were applied: parametric tests (paired and independent sample t-tests) or non-parametric tests (Wilcoxon signed-rank and Chi-squared tests). Relationships between continuous variables were assessed using Pearson correlation.

The assumption of normality was evaluated using the Shapiro–Wilk test, supported by visual inspection through histograms and Q–Q plots. Log transformations were applied where necessary to satisfy test assumptions.

To compare total completion times between DIGM and finger-prick testing, the distribution of paired differences was assessed using the Shapiro–Wilk test, which indicated a significant deviation from normality (W = 0.871, p = 0.0045). A log transformation resolved this (Shapiro–Wilk p = 0.5189), and a paired t-test was performed on the transformed data. In parallel, a Wilcoxon signed-rank test was applied to the original data, returning a significant result (W = 0.0, p < 0.0001). The consistency across both methods supports the robustness of the observed effect.

A *post hoc* power analysis confirmed that the study was sufficiently powered to detect differences between monitoring methods, with a calculated statistical power of 1.000, indicating high statistical sensitivity despite the modest sample size (n = 25).

A p-value < 0.05 was considered statistically significant.

### 2.5 Outcome measurements

The primary outcome measures for this study focused on procedural timing variables and healthcare staff perceptions, obtained through video-recorded simulation tasks and a structured mixed-methods survey.

Timing data were extracted from recordings of participants performing three predefined tasks within a simulated hospital environment: (A) application of the DIGM sensor, (B) retrieval of a glucose reading from a pre-applied DIGM sensor using the FreeStyle Libre device, and (C) capillary blood glucose measurement via traditional finger-prick testing. For each task, specific time intervals (measured in seconds) were recorded, including consent time (duration to obtain consent from the simulated patient), set-up time (preparation of necessary equipment), glucose reading time (execution of the measurement), and total procedural time. The total time encompassed all steps from initiation to completion, including documentation and disposal.

Healthcare staff perceptions were assessed via a structured survey, which included Likert-scale items and one open-ended question. The survey evaluated several dimensions of user experience with DIGM relative to finger-prick methods. Key outcomes included perceived efficiency (time-saving and impact on workflow), infection risk (cross-contamination concerns), and usability (ease of setup, procedural complexity, and overall satisfaction). Additional items explored staff preference and willingness to adopt DIGM in clinical settings. Qualitative responses were analyzed to identify perceived barriers and facilitators to DIGM implementation, such as training needs, device accessibility, patient comfort, and perceived cost-effectiveness.

## 3 Results

**Figure 2:**
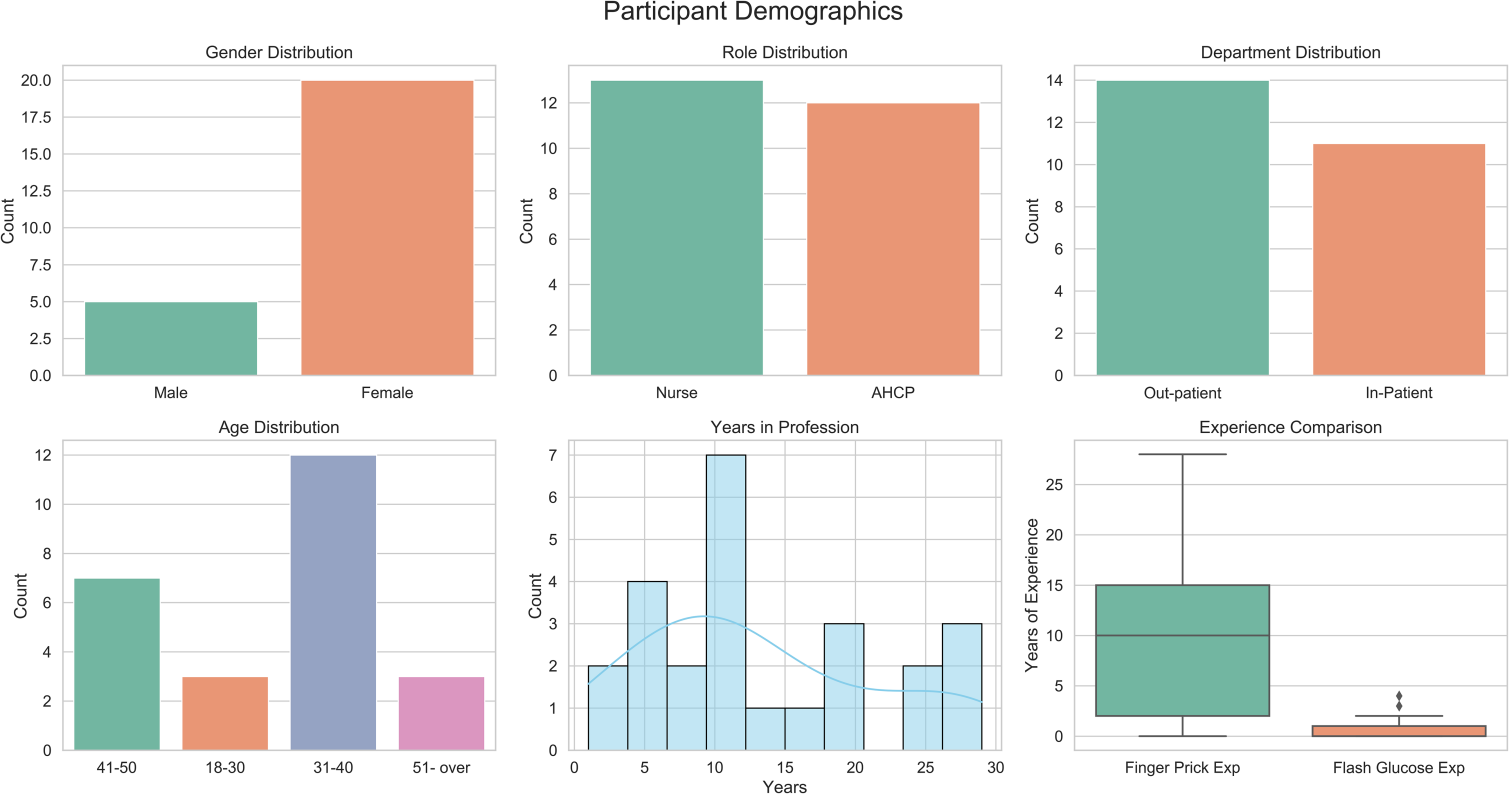
Demographic characteristics of study participants, including professional roles, age distribution, and experience with glucose monitoring.

Of the 25 participants, 80% were female (n = 20), and 20% were male (n = 5). About half were nurses (52%), while 48% were AHCPs. Departmental distribution was balanced, with 44% working in inpatient settings and 56% in outpatient services. Participants had a mean of 13.16 ± 8.56 years of professional experience (1–29 years). Experience with traditional finger-prick glucose monitoring was substantially higher than with DIGM, with a mean of 10.24 ± 8.85 years compared to 0.74 ± 1.16 years, respectively. Regarding age distribution, nearly half of the participants were in the 31–40 age range. A summary of the demographic characteristics is presented in **Figure 2**.

**Figure 3:**
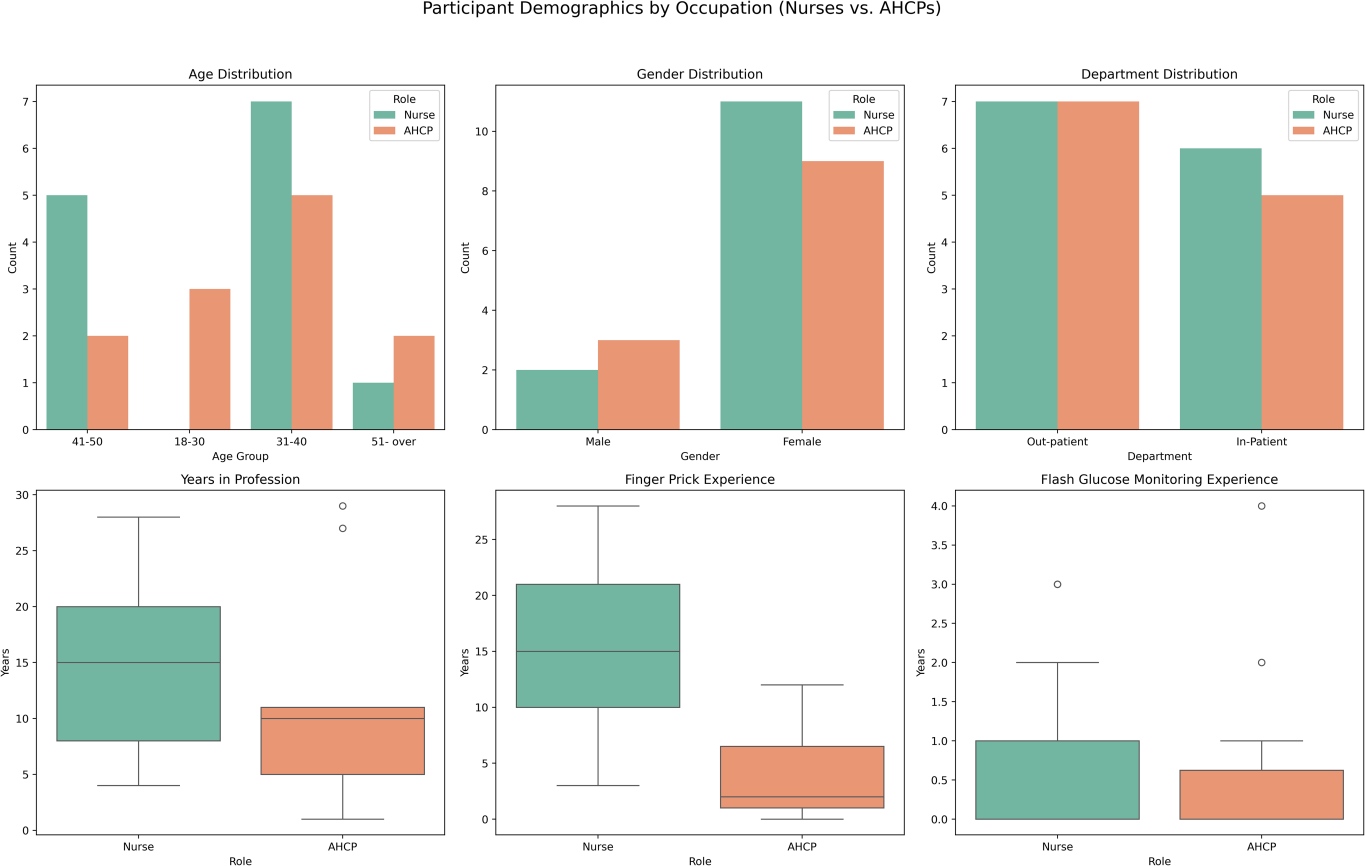
Comparison of participant characteristics (nurses vs. AHCPs), including years of experience and familiarity with glucose monitoring methods.

A comparison of participant demographics by professional role (nurses vs. AHCPs) is presented in **Figure 3**. Most participants in both groups were between 31 and 40 years old. Gender distribution was comparable, with predominating female participants across both cohorts. Departmental affiliation was evenly split between inpatient and outpatient services. Notable differences emerged in professional experience. Nurses reported a longer duration of clinical practice, with a median of over 15 years, compared to a median of approximately 7 years among AHCPs. Additionally, nurses demonstrated greater familiarity with finger-prick glucose monitoring, and both groups had limited prior exposure to DIGM. These findings suggest that while traditional monitoring methods are well established among nursing staff, familiarity with digital glucose monitoring remains limited.

### 3.1 Survey results

**Figure 4:**
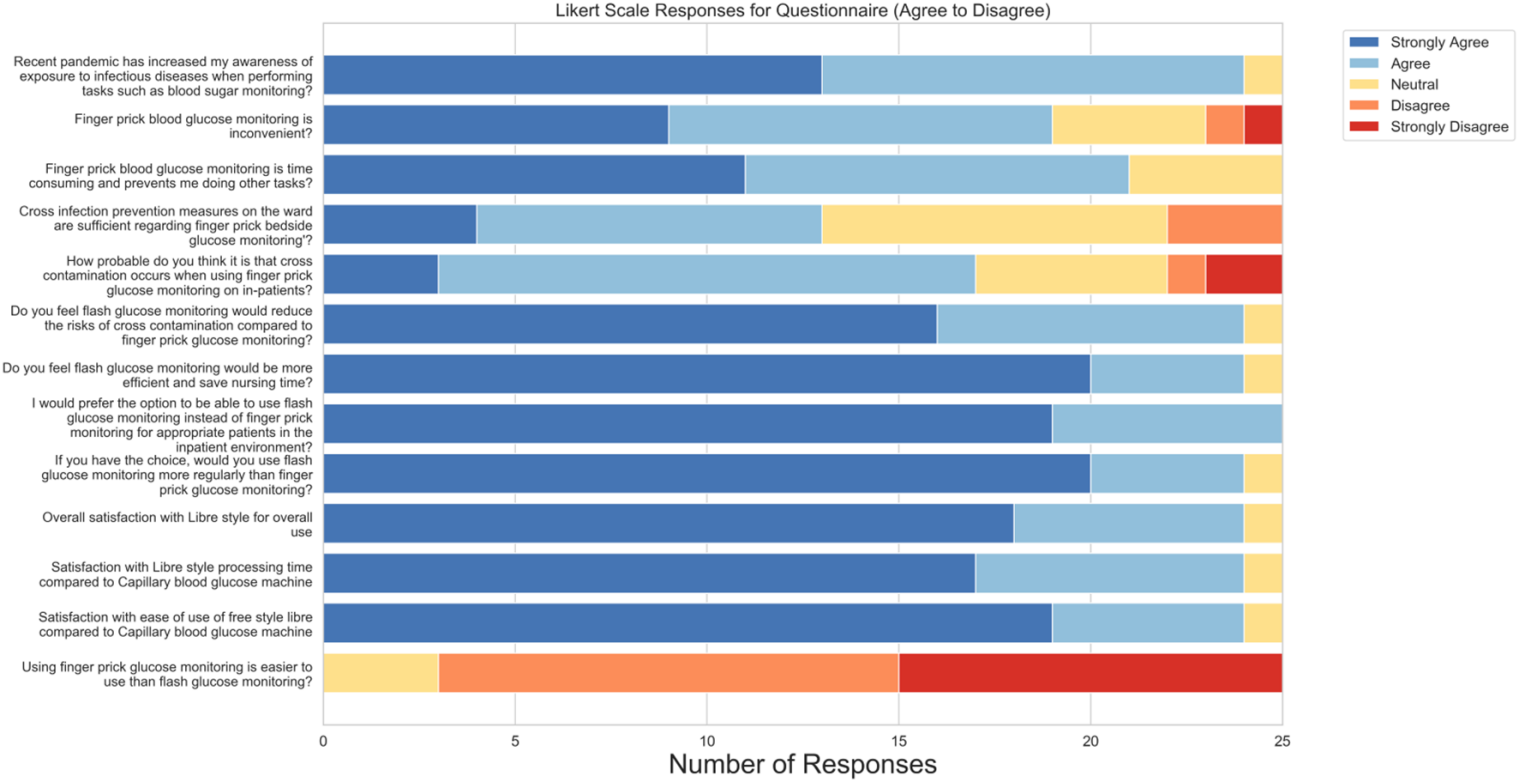
Aggregated responses to Likert-scale survey items assessing healthcare staff perceptions of DIGM versus traditional finger-prick testing.

All 25 participants completed the Likert-scale items, and 22 provided responses to the open-ended questions. **Figure 4** presents the aggregated responses to the Likert items assessing healthcare staff perceptions of DIGM compared to traditional finger-prick methods.

A large majority (96%) agreed or strongly agreed that the COVID-19 pandemic increased their awareness of infection risks during procedures such as blood glucose monitoring, suggesting increased awareness around cross-contamination. Furthermore, 76% of respondents reported finding finger-prick monitoring inconvenient, while 84% perceived it as time-consuming and disruptive to their other clinical responsibilities. These responses reflect the need for more efficient glucose monitoring solutions in routine care.

Concerns regarding infection control were notable. While 68% believed that cross-contamination was likely during finger-prick testing, 48% were neutral or disagreed that existing prevention measures were adequate. In contrast, 96% of participants expressed confidence that DIGM would reduce the risk of cross-contamination, reflecting the technology’s observed safety benefits.

Perceptions of procedural efficiency were similarly positive. 96% agreed that DIGM would save time and be more efficient than finger-prick testing. All respondents (100%) preferred using DIGM over finger-prick methods for patients, and 96% reported adopting it more frequently if available in clinical practice.

User satisfaction with DIGM was high, with 96% indicating they were either satisfied or very satisfied with its overall use. Perceptions regarding ease of use compared to finger-prick testing were more variable: 40% strongly disagreed, 48% disagreed, and 12% were neutral when asked whether finger-prick testing was easier to use than DIGM.

Qualitative responses supported the quantitative findings. Participants highlighted DIGM’s convenience, non-invasiveness, and time-saving potential. Several respondents emphasized the importance of appropriate training to support implementation. Additional themes included perceived improvements in patient comfort, reduced staff burden, and overall cost-effectiveness. Infection control advantages were also frequently cited, reinforcing the technology’s relevance in clinical settings.

These findings indicate strong staff preference, satisfaction, and willingness to adopt DIGM in routine inpatient care, particularly when time efficiency, safety, and workflow integration are essential.

### 3.2 Procedure timings

A detailed comparison of procedure durations between DIGM and traditional finger-prick methods is presented in **Table 1**. Raw mean times (in seconds), standard deviations, and log-transformed values are reported for each paired task. Overall, the findings indicate a significant time-saving potential of DIGM across multiple procedural steps.

**Table 1:** Raw and log-transformed procedure times for DIGM (Tasks A and B) and finger-prick testing (Task C).

Pair	Process	Mean (sec)	N	Std. Deviation	Log Mean	Log SD	Log Diff Mean	Log Diff SD	p-value (log-transformed)
1	A- Consent	10.52	25	4.593	2.27	0.412	0.25	0.434	0.008
1	C- Consent	8.36	25	4.471	2.02	0.44			
2	A- Sensor application	51.52	25	17.333	3.89	0.344	-0.42	0.378	<.001
2	C- Set-up	78.32	25	27.849	4.31	0.335			
3	A- Sensor activation	13.36	25	4.339	2.54	0.314	-0.89	0.477	<.001
3	C- Glucose value	35.4	25	21.366	3.43	0.505			
4	A- Total time	75.4	25	22.364	4.28	0.309	-0.58	0.267	<.001
4	C- Total time	132.84	25	37.018	4.85	0.268			
5	B- Consent	7.8	25	4.34	1.95	0.452	-0.08	0.4	0.352
5	C- Consent	8.36	25	4.471	2.02	0.44			
6	B- Set-up	7.28	25	3.974	1.84	0.58	-2.46	0.583	<.001
6	C- Set-up	78.32	25	27.849	4.31	0.335			
7	B- Glucose value	5.08	25	3.278	1.44	0.628	-1.99	0.841	<.001
7	C- Glucose value	35.4	25	21.366	3.43	0.505			
8	B- Total time	26.4	25	11.504	3.2	0.388	-1.66	0.384	<.001
8	C- Total time	132.84	25	37.018	4.85	0.268			

Each pair compares equivalent procedural steps (e.g., consent, setup, glucose reading, total time). Times are reported as mean and standard deviation (seconds), with corresponding log-transformed values. Mean differences and p-values (paired t-tests) are based on log-transformed data.

#### 3.2.1 Comparison of sensor application and finger-prick glucose measurement

Sensor application (Task A) was significantly faster than finger-prick glucose monitoring (Task C) across all measured components. Consent time was marginally longer for DIGM (10.52 ± 4.59 s) than for finger-prick testing (8.36 ± 4.47 s; p = 0.008). However, setup time for DIGM was substantially shorter (51.52 ± 17.33 s) than that required for finger-prick preparation (78.32 ± 27.85 s; p < 0.001). Similarly, the time to obtain a glucose value was significantly lower for DIGM (13.36 ± 4.34 s) compared to finger-prick testing (35.40 ± 21.37 s; p < 0.001).

The total completion time (consent, setup, measurement, documentation, and waste disposal) was 75.4 ± 22.4 s for DIGM and 132.8 ± 37.0 s for finger-prick monitoring (p < 0.001), highlighting a significant advantage for the digital method (**Table 1**).

#### 3.2.2 Comparison of glucose reading using FreeStyle Libre and finger-prick testing

Obtaining a glucose reading using the FreeStyle Libre device (Task B) was significantly faster than finger-prick testing (Task C). Consent time for Task B was 7.8 ± 4.34 s, compared to 8.36 ± 4.47 s for Task C; however, this difference was not statistically significant (p = 0.352). Setup time was substantially shorter for Task B (7.28 ± 3.97 s) versus Task C (78.32 ± 27.85 s; p < 0.001), and measurement time was also reduced (5.08 ± 3.28 s vs. 35.40 ± 21.37 s; p < 0.001).

The total task duration averaged 26.4 ± 11.5 s for flash glucose reading (Task B), compared to 132.8 ± 37.0 s for finger-prick testing (Task C; p < 0.001), highlighting the procedural efficiency of DIGM (**Table 1**).

#### 3.2.3 Procedure timings by professional role

Timing differences between nurses and AHCPs are summarized in **Table 2**. Nurses required significantly more time than AHCPs for sensor application (85.0 ± 24.4 s vs. 65.0 ± 14.6 s; p < 0.05) and obtaining a glucose reading using the FreeStyle Libre device (31.5 ± 13.6 s vs. 20.8 ± 4.8 s; p < 0.05). However, no statistically significant difference was observed between the two groups in completing finger-prick glucose testing (129.5 ± 31.2 s for nurses vs. 136.5 ± 43.6 s for AHCPs, p = 0.69).

**Table 2:** Comparison of total procedure times between nurses and AHCPs (raw and log-transformed).

Comparison	Nurse Mean (sec)	Nurse SD	Nurse Log Mean	Nurse Log SD	AHCP Mean (sec)	AHCP SD	AHCP Log Mean	AHCP Log SD	p-value (log-transformed)
Apply sensor (A-total time)	85	24.41	4.4	0.33	65	14.64	4.15	0.233	0.04
Glucose reading via sensor (B-Total time)	31.54	13.57	3.37	0.402	20.83	4.76	3.01	0.273	0.014
Finger-prick glucose reading (C-Total time)	129.46	31.17	4.83	0.263	136.5	43.61	4.88	0.283	0.696

Mean times (in seconds), standard deviations (SD), and log-transformed values are shown. Comparisons were performed using independent sample T-tests.

#### 3.2.4 Projected operational time savings and simulation modeling

**Figure 5:**
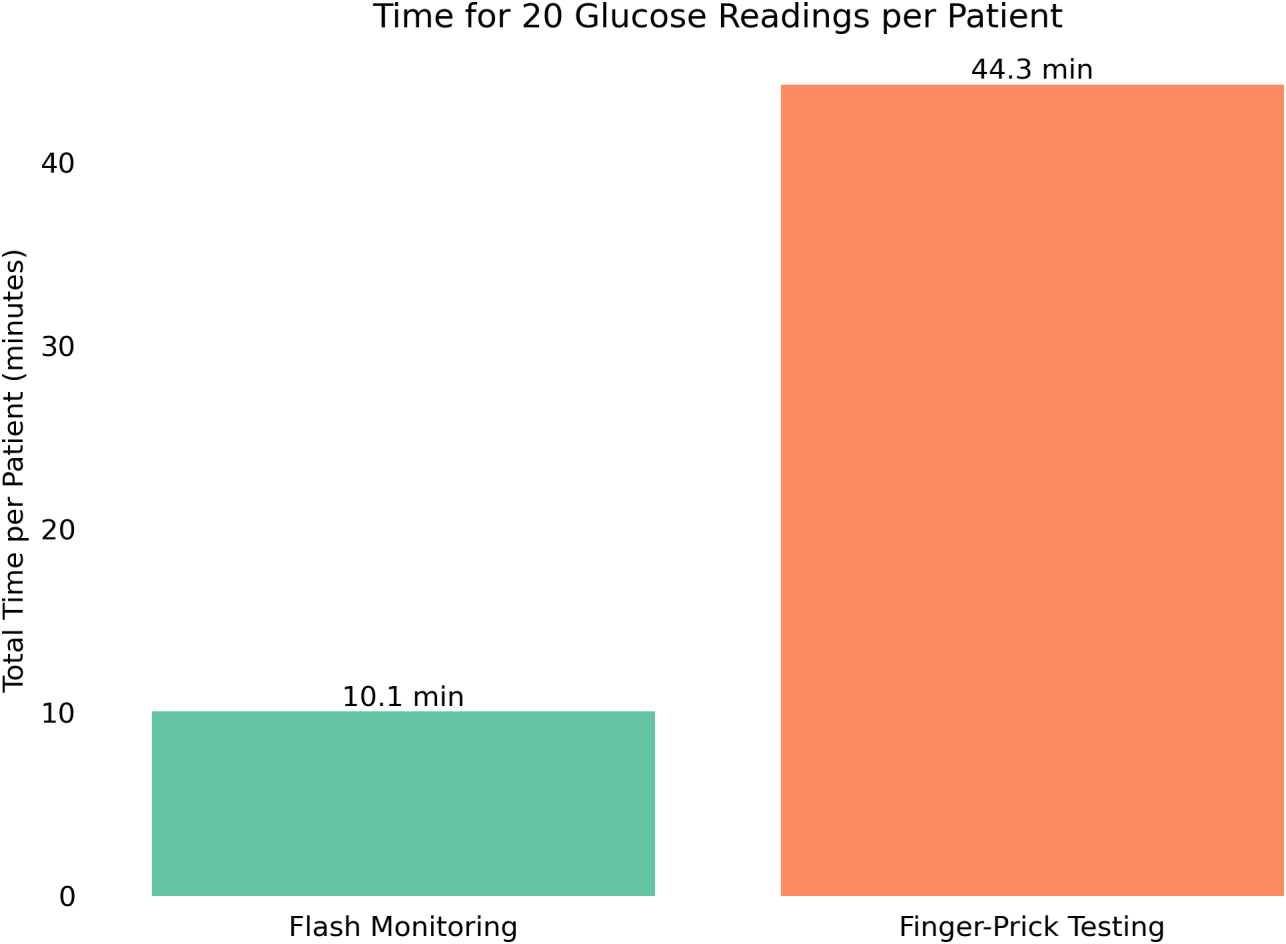
Projected total time required for 20 glucose measurements per patient using either DIGM or finger-prick testing, highlighting time savings with DIGM.

To evaluate the potential operational impact of DIGM in inpatient care, a modeled clinical scenario was developed in which each patient underwent 20 glucose measurements throughout a hospital stay (four measurements per day over an average stay of five days). Based on observed timings, a DIGM cycle, consisting of one sensor application and 20 glucose readings, required approximately 10 minutes of staff time per patient, compared to 44 minutes using the finger-prick method. This represents an estimated time saving of 34 minutes per patient (**Figure 5**).

Extrapolating these findings to a national level, where approximately 1 million patients with diabetes are hospitalized annually in the United Kingdom, the cumulative time savings are substantial. Replacing finger-prick testing with DIGM could save approximately 570,000 hours of staff time, equivalent to more than 65 years of continuous clinical time. These findings suggest that adopting DIGM could significantly alleviate staff workload and improve resource allocation in hospital settings.

**Figure 6:**
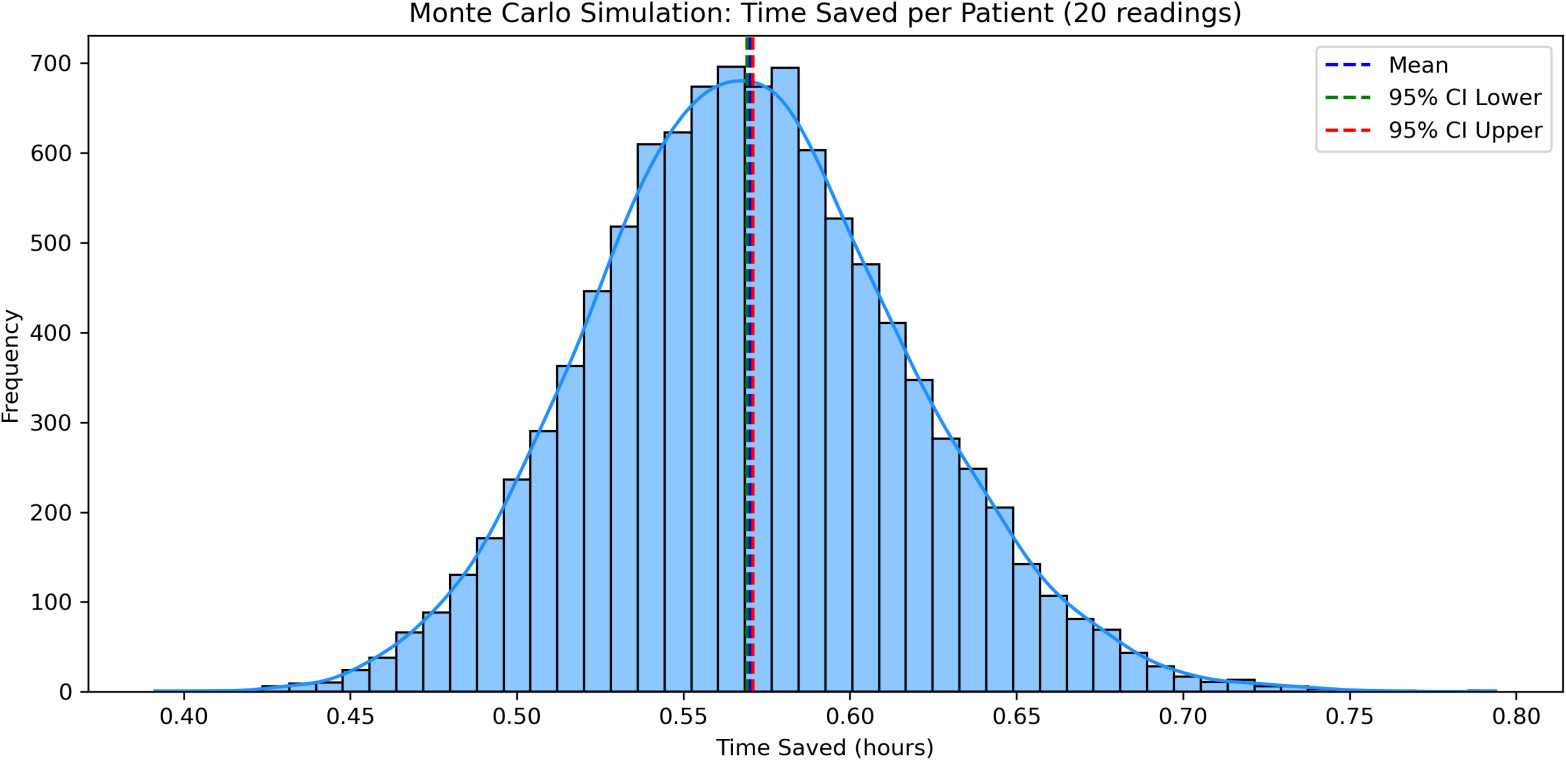
Monte Carlo simulation results demonstrating average staff time saved per patient when using DIGM compared to finger-prick testing.

To validate the robustness of these findings, a Monte Carlo simulation was performed using 10,000 simulated patient scenarios. Each scenario included one sensor application and 20 glucose readings, with task durations randomly sampled from the observed empirical distributions. The simulation returned an average time saving of 33 minutes (0.55 hours) per patient, with a 95% confidence interval ranging from 0.54 to 0.56 hours (**Figure 6**), reinforcing the generalizability of the observed effect.

#### 3.2.5 Relationship between clinical experience and task efficiency

**Figure 7:**
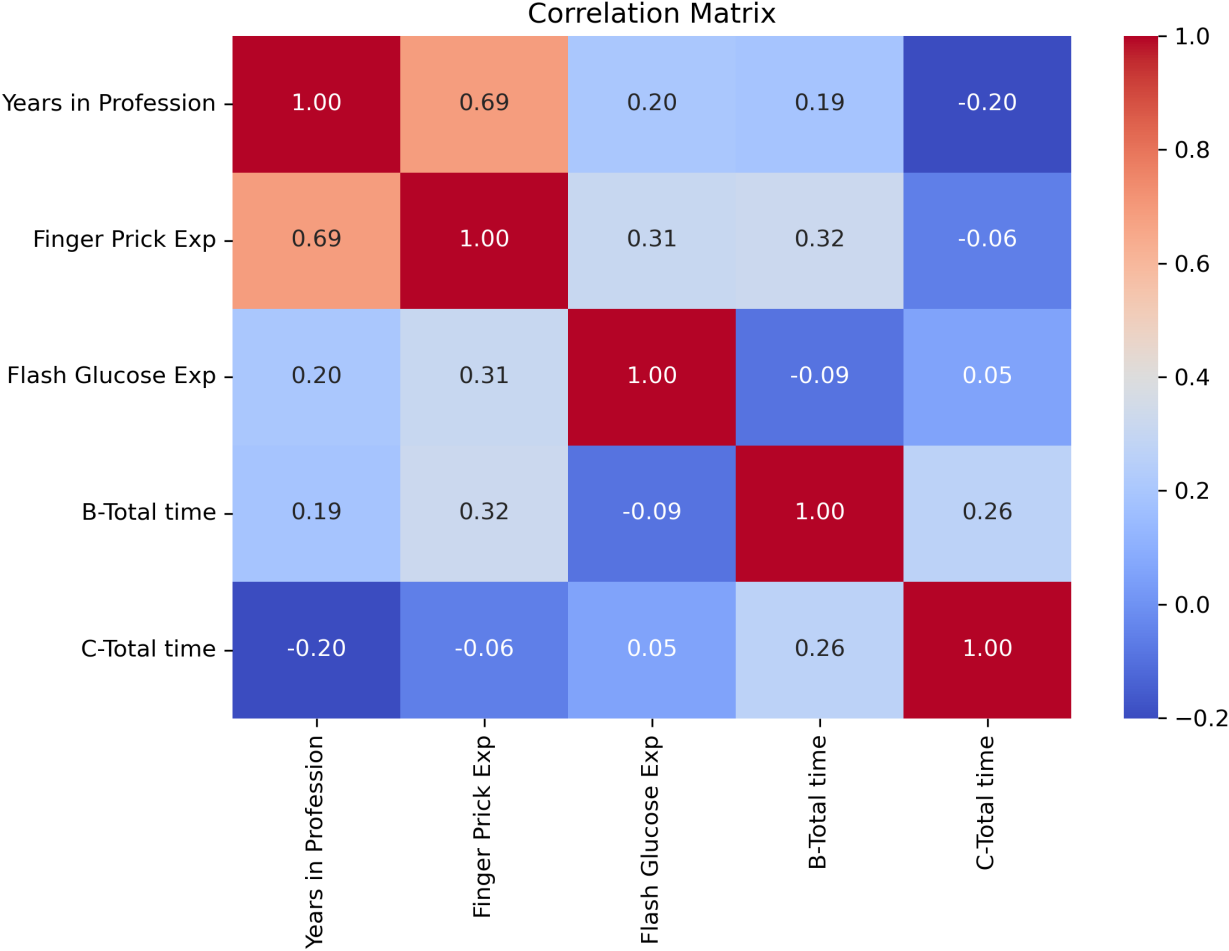
Correlation between professional experience and task efficiency for DIGM and finger-prick glucose monitoring methods.

Pearson correlation analyses were conducted to explore relationships between professional experience and task performance times. A moderate negative correlation was observed between DIGM experience and flash scan times (r = –0.32), as well as between finger-prick experience and finger-prick testing times (r = –0.25) (**Figure 7**), suggesting that increased familiarity may contribute to improved efficiency. Nevertheless, the consistent time-saving benefit of DIGM across all levels of clinical experience highlights its ease of integration into practice.

## 4 Discussion

The simulation-based comparison of DIGM versus traditional finger-prick testing demonstrated significant improvements in workflow efficiency and gathered positive responses from clinical staff.

Participants performed glucose measurements faster using the flash device than finger-prick testing, indicating important time savings per measurement. This suggests that DIGM could streamline hospital workflows, enabling nurses and AHCPs to dedicate more time to other critical patient care tasks. Similar efficiency gains through the adoption of glucose-monitoring technology have been documented in other healthcare settings, particularly regarding workload reduction and improved patient safety (23). For example, a hospital study involving continuous glucose monitoring among 34 inpatients reported savings equivalent to approximately 43 nursing workdays and a substantial reduction in personal protective equipment usage due to fewer fingerstick measurements (18). These efficiency gains underline the operational benefits of integrating DIGM, especially under conditions of resource constraint or heightened clinical demand.

Interestingly, analysis revealed differences in task performance between nurses and AHCPs, highlighting quicker times among AHCPs. These performance differences suggest that tailored training and targeted support may be necessary to optimize the efficiency gains associated with DIGM.

Furthermore, advanced modeling techniques and correlation analyses reinforced DIGM’s efficiency. A Monte Carlo simulation demonstrated consistent average time savings of approximately 33 minutes per patient. Correlation analyses showed improved efficiency with increased familiarity; however, substantial time savings were observed across all experience levels, supporting DIGM’s use in varied clinical settings. These findings are relevant in the context of increasing service demands and workforce pressures within the NHS. Adopting digital health technologies aligns with national priorities outlined in the Topol Review (24), emphasizing innovation’s role in improving productivity and supporting the healthcare workforce. The NHS Change Hub has recently highlighted the continued focus on digital transformation and service improvement (25). By reducing procedural burden and requiring minimal training, DIGM offers a solution to optimize clinical workflow, release staff capacity, and contribute to delivering patient-centered care.

Participant feedback measured through Likert-scale responses indicated a favorable reception towards DIGM. Most nurses and AHCPs described the device as convenient and beneficial for workflow improvement compared to conventional finger-prick methods. This aligns with prior research where DIGM systems have been perceived by clinical staff as user-friendly, accurate, and preferable to traditional testing (23). Staff appreciated the reduced patient discomfort and improved ease-of-use associated with DIGM, which may also enhance patient cooperation. Participants did not encounter significant difficulties operating the device following brief training, implying a manageable learning curve, consistent with findings from prior studies (20, 23).

Infection control emerged as an important consideration. DIGM significantly reduces direct exposure to blood, mitigating risks associated with bloodborne pathogen transmission, compared to finger-prick testing, which involves handling lancets and blood samples. This benefit is consistent with recommendations for inpatient DIGM use to decrease exposure to healthcare staff during infectious outbreaks (26). Participants recognized this advantage, noting the safer, cleaner experience of scanning sensors rather than performing multiple invasive finger-pricks. However, the potential for cross-contamination via shared scanning devices requires effective disinfection protocols or the allocation of dedicated devices per patient, as recommended by infection control guidelines for glucose monitoring equipment (21).

Several limitations of this study should be acknowledged. The relatively modest sample size and single-center recruitment may have introduced selection bias, and staff who volunteered might have been more receptive to new technologies than a general clinical population. Similarly, there is a risk of response bias in the feedback; knowing the study’s purpose, staff may have been inclined (consciously or subconsciously) to provide favorable evaluations of the DIGM. Another consideration is that the order of tasks was not randomized. All participants performed the glucose measurements in a fixed sequence, introducing the potential for order effects. It is possible that familiarity with the simulation scenario or fatigue could have affected performance on the second method, finger-prick testing. Additionally, while controlled and safe, the simulation environment may not fully replicate clinical workflows’ complexities, multitasking demands, and real-time pressures. As such, observed time savings and user experience could differ in clinical practice.

Lastly, our focus on procedural efficiency and user perception meant we did not assess clinical outcomes or sensor accuracy. The flash monitoring system used is a form of intermittently scanned continuous glucose monitoring, which generally provides reliable readings. However, previous studies have reported occasional sensor inaccuracies or data management challenges (27). These considerations underscore that introducing any new monitoring technology requires attention to training, validation, and integration into clinical protocols.

Despite these limitations, this study offers valuable preliminary evidence regarding the potential operational benefits of DIGM in hospital environments. Consistency with existing literature supports the validity and generalizability of these findings. Future research should involve larger, diverse participant samples and real-world clinical settings to confirm the magnitude of observed time efficiencies, evaluate patient outcomes, and identify practical challenges over longer periods.

## 5 Conclusion

In conclusion, the simulation demonstrated that DIGM can substantially improve workflow efficiency and reduce staff burden, and it is positively perceived by clinical staff. DIGM could enhance productivity and safety by reducing invasive procedures and staff exposure. Positive staff attitudes suggest that clinical implementation of DIGM would be well-supported, facilitating integration. These findings support the implementation of DIGM in inpatient care to improve glycemic management, streamline workflow, and reduce occupational risks.

Effective implementation will require attention to infection control measures, staff training, and resource considerations. Further clinical studies are needed to confirm these benefits, assess glycemic and patient-centered outcomes, and develop guidelines for routine hospital use. If validated in practice, replacing or supplementing finger-prick testing with DIGM could significantly increase efficiency, safety, and quality of inpatient diabetes care.

## Data Availability

The raw data supporting the conclusions of this article will be made available by the authors, without undue reservation.

## References

[1] Faulds E Dungan K McNett M Jones L Poindexter N Exline M. Nursing perspectives on the use of continuous glucose monitoring in the intensive care unit. J. Diabetes Sci. Technol. (2023) 17:19322968231170616. doi: 10.1177/19322968231170616 PMC10210097 37081831

[2] Barmanray R Kyi M Worth L Colman P Churilov L Fazio T. Hyperglycemia in hospital: an independent marker of infection, acute kidney injury & stroke for hospital inpatients. J. Clin. Endocrinol. Metab. (2024) 109. doi: 10.1210/clinem/dgae051 38279945

[3] Farrugia Y Mangion J Fava MC Vella C Gruppetta M. Inpatient hyperglycemia: impact on morbidity, mortality and re-hospitalisation rates. Clin. Med. (London). (2022) 22:12–3. doi: 10.7861/clinmed.22-4-s12 PMC9345222 35882487

[4] Umpierrez GE Pasquel FJ. Management of diabetes and hyperglycemia in hospitalized patients. In: Feingold KR, editors. Endotext. MDText.com, Inc, South Dartmouth, MA (2022). (updated 2022).

[5] Leelarathna L Wilmot EG. Flash forward: a review of flash glucose monitoring. Diabetic Med. (2018) 35:472–82. doi: 10.1111/dme.2018.35.issue-4 29356072

[6] Leelarathna L Evans ML Neupane S Rayman G Lumley S Cranston I. Intermittently scanned continuous glucose monitoring for type 1 diabetes. New Engl. J. Med. (2022) 387:1477–87. doi: 10.1056/NEJMoa2205650 36198143

[7] Chagas GSF Torres LMF Clemente MM Lima RCF Pestana DVS Sombrio LS. Use of continuous glucose monitoring and point-of-care glucose testing in hospitalized patients with diabetes mellitus in non-intensive care unit settings: A systematic review and meta-analysis of randomized controlled trials. EClinicalMedicine. 2025 79:101952. doi: 10.1016/j.eclinm.2024.101952 39798897

[8] Castellana M Parisi C Di Molfetta S Di Gioia L Natalicchio A Perrini S. Efficacy and safety of flash glucose monitoring in patients with type 1 and type 2 diabetes: a systematic review and meta-analysis. BMJ Open Diabetes Res. Care. (2020) 8:e001092. doi: 10.1136/bmjdrc-2019-001092 PMC7265013 32487593

[9] Diatribe Foundation. Comparing FreeStyle Libre vs. Finger-sticks in Type 2 Diabetes. San Francisco, California, USA: Diatribe (2024).

[10] Riveline JP Roussel R Vicaut E de Pouvourville G Detournay B Emery C. Reduced rate of acute diabetes events with flash glucose monitoring is sustained for 2 years after initiation: extended outcomes from the RELIEF study. Diabetes Technol. Ther. (2022) 24:611–8. doi: 10.1089/dia.2022.0085 35604792

[11] Evans M Welsh Z Ells S Seibold A. Reductions in HbA1c with flash glucose monitoring are sustained up to 24 months: meta-analysis of 75 real-world studies. Diabetes Ther. (2022) 13:735–53. doi: 10.1007/s13300-022-01253-9 PMC9174370 35476279

[12] Avari P Lumb A Flanagan D Rayman G Misra S Dhatariya K. Continuous glucose monitoring within hospital: A scoping review and summary of guidelines from the joint British diabetes societies for inpatient care. J. Diabetes Sci. Technol. (2023) 17:611–24. doi: 10.1177/19322968221137338 PMC10210120 36444418

[13] The University of Manchester. New study reveals positive impacts of Flash blood glucose monitoring on blood sugar and quality of life. Manchester, England, United Kingdom: Manchester News (2022).

[14] Robbins T Hopper A Brophy J Pearson E Suthantirakumar R Vankad M. Digitally enabled flash glucose monitoring for inpatients with COVID-19: Feasibility and pilot implementation in a teaching NHS Hospital in the UK. Digital Health. (2022) 8:20552076211059350. doi: 10.1177/20552076211059350 35024157 PMC8744149

[15] Longo RR Elias H Khan M Seley JJ. Use and accuracy of inpatient CGM during the COVID-19 pandemic: an observational study of general medicine and ICU patients. J. Diabetes Sci. Technol. (2022) 16:pp. doi: 10.1177/19322968211008446 PMC9445343 33971753

[16] Wollersheim T Engelhardt LJ Pachulla J Moergeli R Koch S Spies C Hiesmayr M Weber-Carstens S. Accuracy, reliability, feasibility and nurse acceptance of a subcutaneous continuous glucose management system in critically ill patients: a prospective clinical trial. Annals of Intensive Care. 2016 6:70. doi: 10.1186/s13613-016-0167-z 27439710 PMC4954792

[17] Avari P Lumb A Flanagan D Rayman G Misra S Dhatariya K Choudhary. Continuous Glucose Monitoring Within Hospital: A Scoping Review and Summary of Guidelines From the Joint British Diabetes Societies for Inpatient Care. Journal of Diabetes Science and Technology. 2023 17(3):611–24. doi: 10.1177/19322968221137338 PMC10210120 36444418

[18] Kazlauskaite R. Remote monitoring is working in the fight against COVID-19. South Brunswick Township, New Jersey, USA: Managed Healthcare Executive (2020).

[19] DeSalvo DJ Lanzinger S Noor N Steigleder-Schweiger C Ebekozien O von Sengbusch S. Transatlantic Comparison of Pediatric Continuous Glucose Monitoring Use in the Diabetes-Patienten-Verlaufsdokumentation Initiative and Type 1 Diabetes Exchange Quality Improvement Collaborative. Diabetes Technol Ther. 2022 24(12):920–4. doi: 10.1089/dia.2022.0248 35947079

[20] Tian T Aaron RE Yeung AM Huang J Drincic A Seley JJ. Use of continuous glucose monitors in the hospital: the diabetes technology society hospital meeting report 2023. J. Diabetes Sci. Technol. (2023) 17:1392–418. doi: 10.1177/19322968231186575 PMC10563530 37559371

[21] Centers for Disease Control and Prevention (CDC). Considerations for blood glucose monitoring and insulin administration. In: CDC Injection Safety Guidelines. Atlanta, Georgia, USA: CDC (2024).

[22] American Diabetes Association (ADA). Consensus considerations and good practice points for continuous glucose monitoring in hospitals. Diabetes Care. (2023) 46:2062–4. doi: 10.2337/dci22-0073 PMC12328255 39452893

[23] Pattison JL Dungan KM Buschur EO Exline M Jone L Faulds ER. (2022). Nursing perspectives on hospital use of Dexcom continuous glucose monitoring in patients with suspected or known COVID-19 during insulin infusion, in: Abstract 67-LB, 82nd Scientific Sessions of the American Diabetes Association, June 2022, Unpublished conference paper.

[24] Topol E. Preparing the healthcare workforce to deliver the digital future: The Topol Review. Leicester, England, United Kingdom: NHS Health Education England (2019). Available online at: https://topol.hee.nhs.uk/ (Accessed April, 4, 2025).

[25] NHS. NHS Change Hub: Driving Improvement Across Health and Care (2025). Available online at: https://change.nhs.uk/en-GB/ (Accessed 10 April 2025).

[26] Frost M. Continuous glucose monitoring gains traction in hospitals. ACP Hospitalist. (2021). Available online at: https://immattersacp.org/archives/2021/03/continuous-glucose-monitoring-gains-traction-in-hospitals.htm.

[27] Sivasubramanian M Avari P Gilbert C Doodson L Morgan K Oliver N Shah P. Accuracy and impact on quality of life of real-time continuous glucose monitoring in children with hyperinsulinaemic hypoglycaemia. Front Endocrinol (Lausanne). 2023 14:1265076. doi: 10.3389/fendo.2023.1265076 37822600 PMC10562688

