# Supplementary material for: Comparing glucose monitoring methods: efficiency insights in a simulated hospital setting
Source: Front Clin Diabetes Healthc. 2025 Jun 3;6:1517161. doi: 10.3389/fcdhc.2025.1517161 (PMC12170324; doi:10.3389/fcdhc.2025.1517161)
Supplement: Supplementary file 1 [file DataSheet1.docx]

**Questionnaire/Survey for Flash Glucose Monitoring for Hospital In-patient**

**About the study**

This study is a research project which is being carried out to see how University Hospital Coventry Staff working in diabetes setting such as ward 1 and Wisdem, perceptions and behaviour using Flash glucose monitoring compared to finger Prick glucose monitoring.

**How your information will be used**

Your personal contact information will not be passed to any third party, this survey will separated be stored securely in line with UHCW data protection protocols. It will not be shared with anyone outside the research team, and you will not be identifiable in any reporting of the survey results.

Participation in this research is entirely voluntary. Completing this survey does not commit you to completing any of the subsequent surveys.

SECTION 1: CONSENT

If you decide to participate in this study, you must first consent to the UHCW Privacy Notice.

I am 18 years old or over.  I consent to the processing of my response, as described in the UHCW Privacy Notice, for the purpose of the research project: Flash Glucose Monitoring for Hospital In-patient. All personal data is processed in accordance with the applicable UK data protection legislation.

**Do you consent to complete the following questionnaire based on the above statements?**

- I Agree
- I Disagree

**Please Select: AHCP
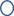
 Nurse
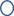
**

**Q1. How old are you?**


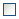
 18 - 30


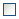
 31 - 40


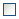
 41 – 50


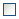
 51 or over

**Q2. What gender are you?**


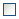
 Male


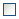
 Female


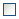
 Prefer not to say

**Q3. Which department you work:** In-patient
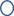
 Outpatient
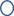


**Q4. Years in profession: _____**

**Q5. Capillary blood glucose experience/finger prick in years or months: _____**

**Q6. Flash Glucose Monitoring experience in years or months: _____ (or none)**

***Staff Habits***

**Q7. How strongly do you agree with the following statement “Recent pandemic has increased my awareness of exposure to infectious diseases when performing tasks such as blood sugar monitoring?”**


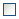
 Strongly Agree


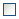
 Agree


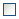
 Neutral


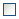
 Disagree


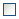
 Strongly Disagree

***Staff Perceptions & safety***

**Q8.** **How strongly do you agree with this statement “Finger prick blood glucose monitoring is inconvenient”?**


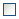
 Strongly Agree


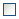
 Agree


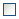
 Neutral


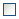
 Disagree


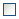
 Strongly Disagree

**Q9. How strongly do you agree with this statement “Finger prick blood glucose monitoring is time consuming and prevents me doing other tasks”?**


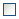
 Strongly Agree


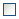
 Agree


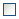
 Neutral


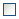
 Disagree


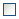
 Strongly Disagree

**Q10. How strongly would you agree with the statement “cross infection prevention measures on the ward are sufficient regarding finger prick bed side glucose monitoring?”**


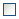
 Strongly Agree


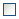
 Agree


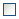
 Neutral


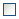
 Disagree


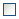
 Strongly Disagree

**Q11. How probable do you think it would be cross contaminated when using finger prick glucose monitoring on in-patient?**


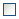
 Very Probable


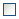
 Probable


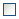
 Somewhat Probable


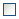
 Neutral


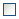
 Not Probable

**Q12. Do you feel flash glucose monitoring would reduce the risks of cross contamination (or exposure to infectious patients) compared to finger prick glucose monitoring?**


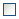
 Very Confident


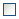
 Confident


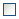
 Not too Confident


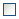
 Not Confident at All


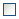
 No Change

**Q13. Do you feel flash glucose monitoring would be more efficient and save nursing time?**


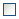
 Very Confident


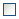
 Confident


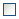
 Not too Confident


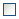
 Not Confident at All


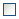
 No Change

**Q14. How strongly do you agree with the statement “I would prefer the option to be able to use flash glucose monitoring instead of finger prick monitoring for appropriate patients in the inpatient environment”**


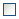
 Strongly Agree


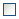
 Agree


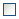
 Neutral


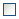
 Disagree


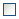
 Strongly Disagree

***Overall Satisfaction***

**Q15. If you have the choice, would you use the flash glucose monitoring more regularly than finger prick glucose monitoring?**


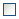
 Use much more often


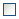
 Use more often


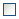
 No change


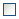
 Use less


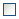
 Use much less

**. Time and ease of use**

|  | Very satisfied | Satisfied | Neutral | Unsatisfied | Very unsatisfied |
| --- | --- | --- | --- | --- | --- |
| 16. Overall Use of Libre style |  |  |  |  |  |
| 17. Libre style processing time compared to Capillary blood glucose machine |  |  |  |  |  |
| 18. Ease of use of free style libre compared to Capillary blood glucose machine |  |  |  |  |  |

**Q19. How strongly do you agree with this statement “Using Finger prick glucose monitoring is easier to use than Flash glucose monitoring”?**


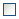
 Strongly Agree


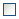
 Agree


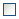
 Neutral


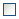
 Disagree


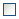
 Strongly Disagree

**Q20. What do you feel would be the most important thing to encourage you to use flash glucose monitoring more often?**

………………………………………………………………………………………………………………………………………………………………………………………………………………………………………………………………………………………………………………………………………………………………………………………………………………………………………………………
